# Study of Protein Expresion in Peri-Infarct Tissue after Cerebral Ischemia

**DOI:** 10.1038/srep12030

**Published:** 2015-07-08

**Authors:** David Brea, Jesús Agulla, An Staes, Kris Gevaert, Francisco Campos, Tomás Sobrino, Miguel Blanco, Antoni Dávalos, José Castillo, Pedro Ramos-Cabrer

**Affiliations:** 1 Neurology Department, Neurovascular Area, Clinical Neurosciences Research Laboratory, University Clinical Hospital, Health Research Institute of Santiago de Compostela (IDIS), University of Santiago de Compostela, Spain; 2 Cellular and Molecular Neurobiology Research Group and Grup de Recerça en Neurociencies del IGTP, Department of Neurosciences, Fundació Institut d’Investigació en Ciències de la Salut Germans Trias I Pujol-Universitat Autónoma de Barcelona, Badalona, Spain; 3 Research Unit, University Hospital of Salamanca and Institute of Health Sciences of Castilla and Leon, Salamanca, Spain; 4 Department of Medical Protein Research, VIB, Ghent, Belgium; 5 Department of Biochemistry, Ghent University, Ghent, Belgium

## Abstract

In this work, we report our study of protein expression in rat peri-infarct tissue, 48 h after the induction of permanent focal cerebral ischemia. Two proteomic approaches, gel electrophoresis with mass spectrometry and combined fractional diagonal chromatography (COFRADIC), were performed using tissue samples from the periphery of the induced cerebral ischemic lesions, using tissue from the contra-lateral hemisphere as a control. Several protein spots (3408) were identified by gel electrophoresis, and 11 showed significant differences in expression between peri-infarct and contra-lateral tissues (at least 3-fold, p < 0.05). Using COFRADIC, 5412 proteins were identified, with 72 showing a difference in expression. Apart from blood-related proteins (such as serum albumin), both techniques showed that the 70 kDa family of heat shock proteins were highly expressed in the peri-infarct tissue. Further studies by 1D and 2D western blotting and immunohistochemistry revealed that only one member of this family (the inducible form, HSP72 or HSP70i) is specifically expressed by the peri-infarct tissue, while the majority of this family (the constitutive form, HSC70 or HSP70c) is expressed in the whole brain. Our data support that HSP72 is a suitable biomarker of peri-infarct tissue in the ischemic brain.

Stroke is the second leading cause of death, the second leading cause of dementia, and the leading cause of major disability in adults, often requiring institutional care due to loss of independence^1^. Stroke consists of the loss of cerebral functions resulting from the interruption of blood supply to a region of the brain. When an interruption is not resolved within a proper time, the brain tissue is subjected to a wave of deleterious processes that propagate from the ischemic core (irreversibly damaged tissue) into the surrounding, less severely affected tissue, worsening the clinical outcome of the patient. The latter portion of tissue, generally referred to as the “ischemic penumbra”, is a therapeutic target for the treatment of stroke^2^.

To date, thrombolysis (pharmacological or surgical removal of the obstruction) is the most effective treatment for ischemic stroke at very early stages (3–4.5 h). Beyond this therapeutic window, this procedure usually presents limitations and complications, eventually leading to death^3^. Alternatively, therapeutic strategies based on the pharmacological protection of the penumbra tissue (neuroprotective therapies) have also been proposed, with promising results at preclinical level, but systematically failing in their translation to the clinics for different reasons^4^,^5^.

Recannalization and neuroprotective approaches rapidly loose effectiveness with time, and may be inadequate or ineffective when started at delayed times from the onset of stroke. However, other therapeutic approaches, such as those aimed at enhancing regenerative processes (either endogenous and/or exogenous) and functional reorganization or brain plasticity processes, are usually started 24–72 h post stroke, when the lesion has reached a mature stage and non-conventional therapeutic approaches find more suitable environmental conditions for success^6^,^7^.

At such delayed time-points from the onset of stroke, the conventional radiological markers used to define the penumbra (diffusion and perfusion imaging mismatch) disappear^2^. In this context, it has been suggested that the therapeutic focus should be placed on the so-called “peri-infarct” tissue^6^,^7^, which corresponds to the portion of tissue that surrounds the matured lesion core but, unlike the classical concept of the ischemic penumbra, must be defined by means of specific biomarkers instead of radiological parameters.

In the 90 s, a considerable amount of pre-clinical stroke research was dedicated to studying gene expression changes in the ischemic brain. Heat shock proteins, together with c-fos and junB were some of the first targets to be identified^8^. Some other studies have reviewed gene expression in the ischemic brain, including peri-infarct tissue^9^. However, all of these studies focused on gene expression changes, and this is no guarantee that the protein is produced in the tissue. We wanted to focus our study on proteins rather than gene expression. In this regard, we have found a few studies in the current literature that report the up-regulation of some proteins in the peri-infarct area, such as Beclin-1^10^, SDF-1^11^, or HSP70^12^,^13^, but only predefined proteins have been studied, likely based on those previous works of gene expression. Other authors have performed full proteomic studies of the whole brain or have compared complete brain hemispheres, without discriminating between the lesion core, peri-infarct tissue and unaffected tissue^14^,^15^,^16^,^17^. Therefore, we believe that a global study of the total protein expression from the ischemic brain tissue that is particularly focused on the peri-infarct area would add important information to the field by comparing the potential of different biomarkers for defining (and potentially targeting) this specific and key portion of the brain tissue.

In short, we report here a proteomic analysis of ischemic brain tissue, with a particular focus on the peri-infarct tissue, in an animal model of focal permanent ischemia using two proteomic approaches (2D gel electrophoresis, and a gel-free proteomic technique) complemented with western blotting and immunohistochemical studies. The ultimate aim of this study is to perform a proteomic study of the peri-infarct tissue in order to find robust markers of this key area of the ischemic brain.

## Results

**Figure 1:**
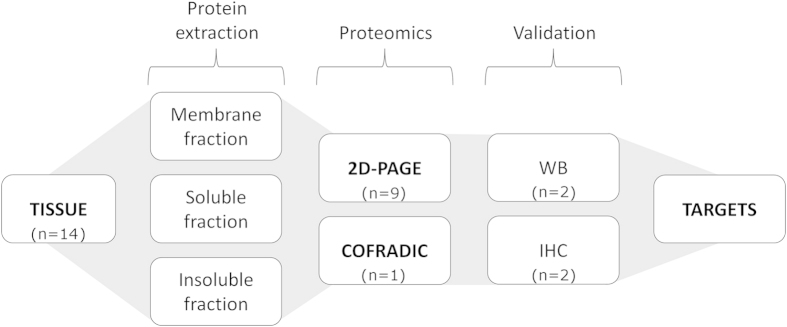
Schematic representation of the experimental procedures performed. Three tissue portions were isolated from rat brains at 48 h post-ischemia: infarcted tissue (lesion), as delimited by TTC staining; a 2-mm strip around the infarct region (peri-infarct); and control tissue from the contra-lateral hemisphere. The protein content from each portion was extracted in three different fractions (membrane, soluble and insoluble). The samples were analysed 2D-PAGE and COFRADIC proteomics. Proteins with significant differences of expression between the tissue portions (>3-fold, p < 0.05) were further studied by western blotting (WB) and immunohistochemistry (IHC). Data were combined to define suitable molecular targets of the peri-infarct tissue.

The experimental procedure used in this study is schematically represented in Fig. 1. Brain sections from animals subjected to a focal permanent cerebral ischemia were subsequently studied by proteomics, blotting and immunohistochemistry techniques, as described in the methods section.

### 2D-PAGE proteomic study

**Figure 2:**
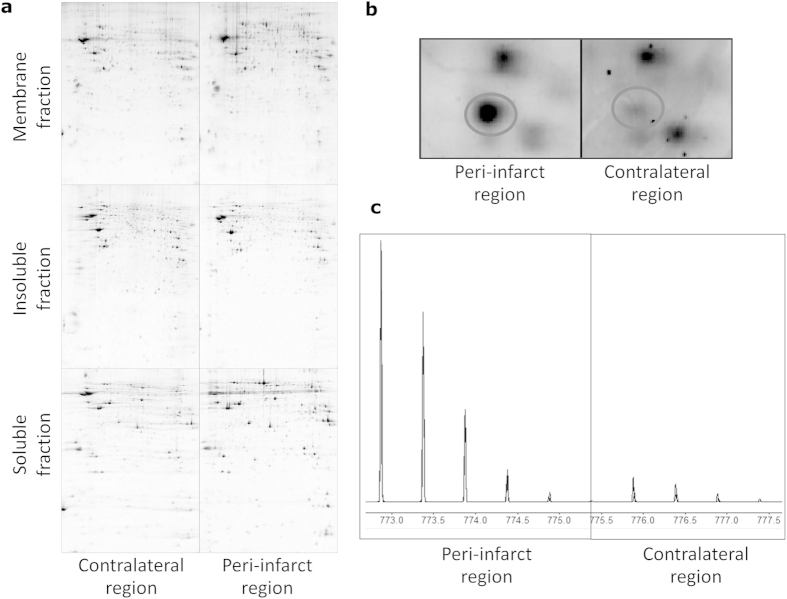
Proteomic studies. **A)** Representative 2D-PAGE gels for the three different protein fractions (membrane, soluble and insoluble fractions) of the control (contra-lateral hemisphere) and peri-infarct tissue. **B)** Magnification of a region of the gels showing the over-expression of a protein (HSP beta-1, in the circle) at the peri-infarct tissue. **C)** Quantitative differences in the expression of ^12^C- (peri-infarct tissue) and ^13^C- (contra-lateral tissue) labelled propionate peptides in the COFRADIC study. Areas under the curve are representative of the levels of expression of the corresponding proteins.

Scanned images of six representative polyacrylamide gels obtained from 2D-PAGE experiments are presented in Fig. 2A (peri-infarct *vs.* contra-lateral tissues and membrane, soluble and insoluble fractions of proteins). Each dark spot in the gels represents a protein or a fraction of a protein. A magnification of the region corresponding to heat shock proteins in the gels from the membrane fraction is presented in Fig. 2B. The stronger staining in the peri-infarct gel represents a higher level of expression of the corresponding protein in this tissue (circled spot) compared to the contra-lateral tissue.

In the 2D-gel analysis, 962 spots were observed for membrane proteins (Fig. 2A), with high reproducibility between gels of the contra-lateral vs. peri-infarct regions (r^2^ = 0.92). No qualitative differences between the peri-infarct and contra-lateral tissues were found, but significant (p < 0.05) quantitative differences (three-fold or higher expression in peri-infarct tissue) were observed for 4 protein spots. Three of those spots corresponded to Serum Albumin, while the fourth corresponded to Serotransferrin.

For the insoluble protein fraction we observed an average of 1632 spots (Fig. 2A), with high reproducibility between gels of the contra-lateral and peri-infarct regions (r^2^ = 0.92). Quantitative analysis of the 2D gels revealed statistically significant differences between peri-infarct and contra-lateral regions for 9 spots (p < 0.05, ≥3-fold over-expressed in the peri-infarct region). These protein spots were identified as Eukaryotic Translation Initiation Factor 2, Tubulin-Beta 2C chain, Heat Shock Protein Beta-1 (two spots), Prohibitin, Actin Cytoplasmic 1, Serum Albumin (two spots) and the 70 kDa Heat Shock Protein.

The soluble protein faction gels contained an average of 814 spots (Fig. 2A), with 94% reproducibility. In this fraction, five spots were over-expressed in the peri-infarct region using the same criteria as before. These spots were identified as Chain A, rat transthyretin complex with thyroxine (T4), dihydropyrimidinase, and dihydropyrimidinase-related protein 2 (3 spots).

**Figure 3:**
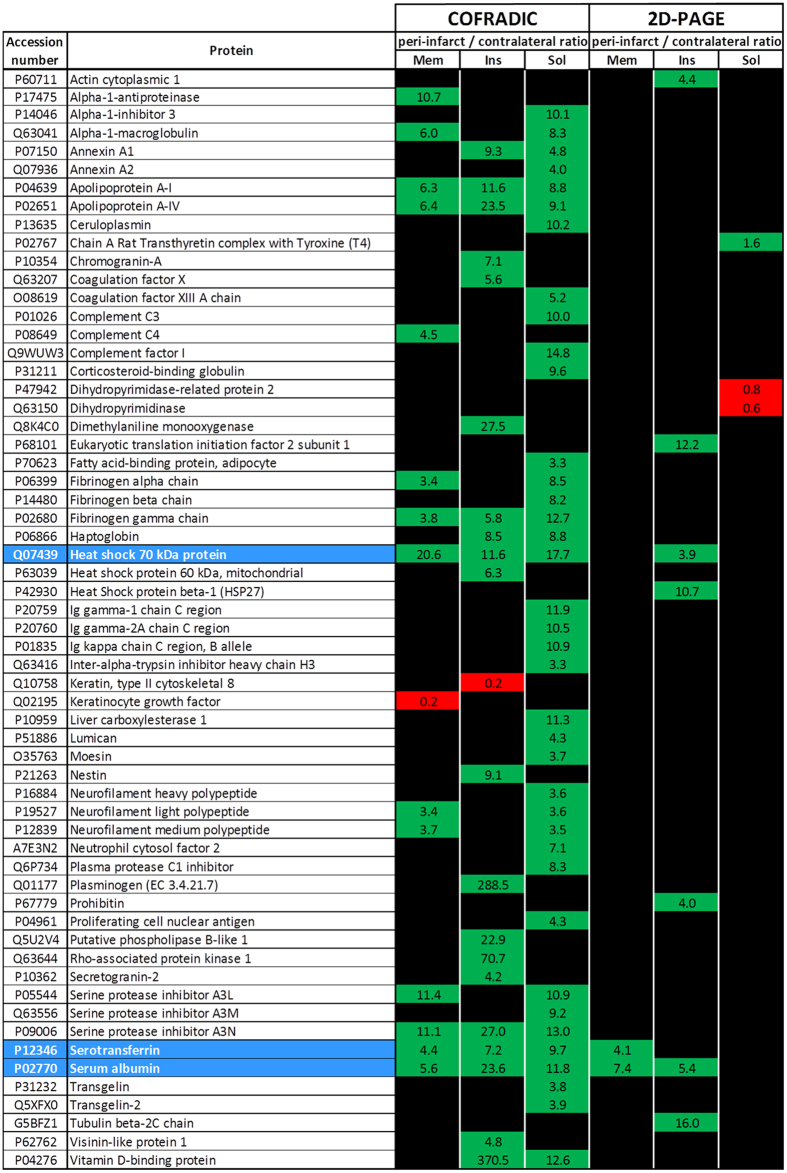
Results from the proteomic analysis of the ischemic brain using 2D-PAGE and COFRADIC techniques. The observed differences in protein expression in the peri-infarct vs. control tissue from the ischemic brain are listed in alphabetical order. Over-expression in the peri-infarct tissue is presented in green boxes, while lower expression in this tissue is presented in red boxes. Numbers in the boxes represent the peri-infarct/contra-lateral ratios. The results for the membrane (mem), soluble (sol) and insoluble (ins) protein fractions are presented in separated columns. Coincident results for both techniques are highlighted in blue.

The results from this study are summarized in Fig. 3, where we show the differences in expression of the proteins found in each protein fraction from either of the two proteomic approaches performed. For each protein, the peri-infarct /contra-lateral ratio of expression is presented in green boxes, when the corresponding protein is over-expressed in the peri-infarct region, or in red boxes, when the protein exhibits a higher level of expression in the contra-lateral region.

### Gel-free COFRADIC proteomic study

COFRADIC studies were performed on all three protein fractions from an ischemic brain. A large number of proteins (1358) were identified in the membrane fraction. Twelve proteins were selected because they exhibited an at least 3-fold difference in expression (see example in Fig. 2A and a complete list in Fig. 3); one protein was down-regulated in the peri-infarct region and the remaining proteins were over-expressed in this region.

We identified 2070 proteins in the insoluble fraction. Among them, one protein was down-regulated and 20 proteins were up-regulated in the peri-infarct region (Fig. 3).

We identified 1984 proteins in the soluble fraction. From this group, 39 showed differential expression between the peri-infarct and contra-lateral regions; all of the proteins were up-regulated in the peri-infarct region (Fig. 3).

We have found three coincidences between the two proteomic approaches used in this study (marked in blue in Fig. 3). Two of them correspond to plasma-related proteins (Serotransferrin and Serum Albumin) and the third corresponds to the 70 kDa family of Heat Shock Proteins (Fig. 3), which showed 20.6-fold (membrane fraction), 11.6-fold (insoluble fraction) or 17.7-fold (soluble fraction) higher levels of expression in the peri-infarct tissue, respectively, according to COFRADIC, and 3.9-fold higher expression in the insoluble fraction of proteins at the peri-infarct tissue, according to 2D-PAGE.

### Western blot studies

**Figure 4:**
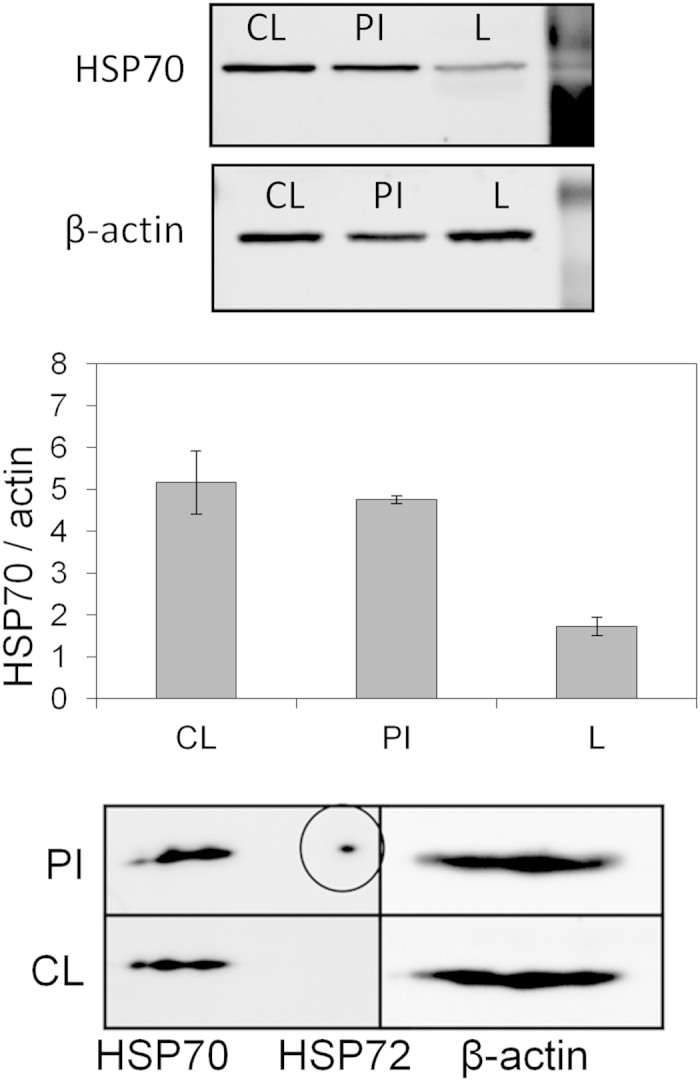
Western blot (WB) analysis of the expression of the 70 kDa Heat Shock Protein (HSP70) in the ischemic brain. **Top)** Cropped band of the WB gel corresponding to HSP70 in control tissue (CL, contra-lateral hemisphere), peri-infarct (PI), and the core of the ischemic lesion (L) from the 1D western blot (isoelectric point). All 3 samples were run together in the same gel. β-Actin was used as the loading control (cropped band shown) in the same gel and at the same time (complete gels presented in supplementary data 1). **Middle)** Quantification of the expression levels, presented as the HSP70/Actin ratio (mean ± SDEV of pooled data from two different rats). **Bottom)** Cropped band corresponding to the second dimension of the western blot study over 2D electrophoresis gels (separation by molecular weight from right to left) for the HSP70 kDa band, showing that a small fraction of this family of proteins (HSP72, HSP70i or inducible form of HSP70, in the circle) is exclusively expressed in the peri-infarct tissue (PI), while a large fraction of this family (constitutive form of HSP70 or HSP70c) is expressed in both the peri-infarct (PI) and the contra-lateral (CL) tissue. All samples were run together in the same gel.

We performed a conventional Western blot analysis of the expression of the 70 kDa family of heat shock proteins from peri-infarct (PI), contra-lateral (CL) and lesion-core (L) tissue samples (Fig. 4, top and supplementary data 1), and separated the proteins according to their molecular weight. β-Actin was used as protein loading control. When the levels of expression of the HSP70 protein were corrected by β-Actin (Fig. 4, middle), we observed no significant differences in the expression between the contra-lateral tissue (HSP70/β-Actin ratio of 5.1) and the peri-infarct tissue (HSP70/β-Actin ratio of 4.8), which were both significantly higher than the expression of HSP70 at the core of the lesion (HSP70/β-Actin ratio of 1.9). Similar results were obtained for other tested proteins that showed significant differences in expression in 2D-PAGE studies, such as dihydropyrimidase-related protein 2 (DHRP-2), eukaryotic translation initiation factor 2 (eIF2α), HSP27, prohibitin and β -tubulin (data not shown).

When a 2D (isoelectric point vs. molecular weight) western blot study was performed for these proteins, we observed that the corresponding spot for the HSP70 family was separated in two different fractions, with the smallest fraction specifically present in the peri-infarct region, and absent in the contra-lateral (control) tissue (Fig. 4, bottom and supplementary data 1). The differential spot was identified as the inducible form of the HSP70 protein family (also named HSP72 or HSP70i), while the common spot for both tissues (peri-infarct and control) was identified as the constitutive form of the HSP70 family (also known as HSC70 or HSP70c)^18^. In all of these studies, β-actin was used as protein loading control. The blot presented at the bottom of Fig. 4 and supplementary data 1 was performed using 2D-electrophoretic gels, and therefore, spots are expected instead of bands as is shown in Fig. 3. Thus, for HSP70 3, spots can be identified that correspond to different post-translational modifications of the protein. In the actin area, at least four spots (postraductional modifications of this protein) can be identified. When the spots are very intense and close to each other, they may look like a band, but in the case of HSP72, only one spot is present and this effect is not observed. The selective presence of any fraction in the peri-infarct tissue was not observed for the other tested proteins, such as dihydropyrimidase-related protein 2 (DHRP-2), eukaryotic translation initiation factor 2 (eIF2α), HSP27, prohibitin or β -tubulin (data not shown).

### Immunohistochemistry studies

**Figure 5:**
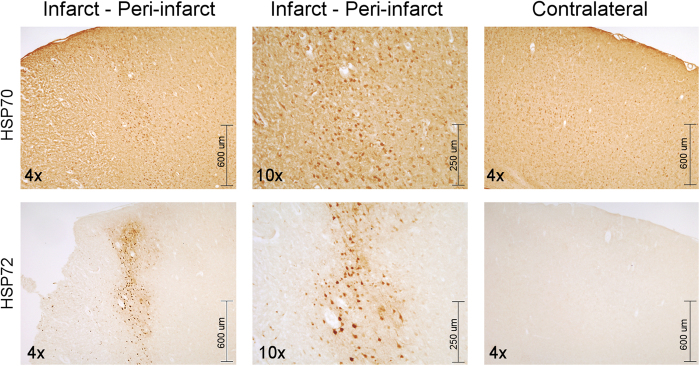
Immunohistochemistry of two consecutive slices of brain tissue showing the expression of HSP70 proteins. Images (4x and 10x) showing the expression of the constitutive form of HSP70 (HSP70 or HSP70c) in cells of the infarct, peri-infarct, and contralateral areas of an ischemic brain (top row), while the inducible form of this protein (HSP72 or HSP70i) is only expressed in cells of the peri-infarct region, and not in the other two regions (lower row).

Immunolabelling of brain sections was performed with an antibody that specifically labelled HSP72 and with another antibody that labelled both the inducible and constitutive forms of HSP70 (Fig. 5). While HSP72 expression was not observed in the contra-lateral tissue and was nearly absent in the lesion site, the expression of this protein is clearly visible in the peri-infarct region. On the contrary, strong staining for the antibody against the constitutive form of HSP70 was observed in both the peri-infarct and the contra-lateral (control) tissue.

## Discussion

Proteomics is a term used to define a series of experimental procedures performed for the identification and quantification of complex protein mixtures. Thus, one can reveal the role of specific proteins during the development of pathological processes. The identification of proteins that play a key role in the progression of a pathology may provide biomarkers/targets for the development of diagnostic and therapeutic approaches to treat the disease being studied.

Here, we have used proteomics to analyse the expression of proteins from different regions of the rat brain at 48 h after the induction of a focal ischemia. Indeed, the definition of suitable molecular markers of the peri-infarct tissue in ischemia that may be potentially used as targets is expected to open new therapeutic opportunities for patients who, unfortunately, could not have been successfully treated with thrombolytic therapies^6^,^7^.

Our main proteomic study was performed using 2D polyacrylamide gel electrophoresis (2D-PAGE), combined with mass spectrometry, in which a complex mixture of proteins are loaded on a polyacrylamide gel and separated depending on their isoelectric point (one dimension) and molecular weight (2 dimensions) by applying electrical currents to the gel. This process is combined with mass spectrometry (MS) for proper identification of selected proteins. 2D-PAGE-MS is considered the gold-standard technique for proteomics, and its validity for identifying targets in the neurosciences is well-documented^19^,^20^,^21^.

Despite the fact that 2D-PAGE has been used for more than 30 years, some reports have suggested that this technique presents the disadvantage of not being suitable for the detection of proteins with low copy numbers (<1,000 copies per cell), which may remain undetected among the more abundant housekeeping and structural genes. In addition, hydrophobic integral membrane proteins are sometimes difficult to extract from the membranes and tend to precipitate easily, especially near their isoelectric point, and their detection is quite difficult by 2D-PAGE^22^. Thus, we complemented our proteomic studies by testing the suitability of a complementary proteomic technique, which is not affected by the previously described drawbacks.

COFRADIC (COmbined FRActional DIagonal Chromatography) is a gel-free chromatographic approach. This fact, together with the use of very sensitive detection of isotopically labelled peptides, allows the detection of proteins with low copy numbers or those that are difficult to isolate^22^,^23^. On the contrary, because only certain peptides are labelled in COFRADIC (methionine-containing peptides in our case), peptides that do not contain this amino acid are not detectable. To solve this problem, one can use other variants of COFRADIC, which analyse peptides containing other amino acids (see Ref. [22]. for a complete description of this technique).

In general, 2D-PAGE and COFRADIC techniques must be considered complementary rather than competitive proteomic approaches, and a combined analysis like the one performed here (summarized in Fig. 4) gives us a more complete picture of the protein expression of the peri-infarct tissue.

Our 2D-PAGE studies revealed that 11 proteins were over expressed in the peri-infarct region, with respect to the brain tissue of the contra-lateral hemisphere (control). However, COFRADIC showed 72 differences in expression in the peri-infarct region, showing a high degree of sensitivity of this technique. The detection of a higher number of differential proteins by COFRADIC may be due to the presence of a greater number of low copy number proteins, which limits their detection by 2D-PAGE (as discussed earlier).

Three coincidences were found between both techniques, which corresponded to the 70 kDa family of heat shock proteins and blood-related proteins (Serotransferrin and Serum Albumin).These results are not surprising, and other authors have also published apparently low coincidences between gel-based and gel-free proteomic analyses^24^.

Proteins with three-fold or higher differences in protein expression in the 2D-PAGE analysis were further studied by 1D western blot (separation by molecular weight), excluding results corresponding to blood-related proteins (such as Serotransferrin and Serum Albumin). For these studies, samples of peri-infarct, lesion core, and contralateral tissues were extracted and processed. In our first approach, we found that there were no significant differences in levels of HSP70 expression between the peri-infarct and the contralateral tissues (Fig. 4 and supplementary data 1), as may be expected from the 2D-PAGE proteomic study. However, it has been reported in the literature that “the inducible (hsp72) and constitutive (hsc70) members of the heat shock 70 gene family are highly homologous, necessitating development of techniques that accurately identify and measure constitutive and inducible members”^25^.

Additionally, proteins are not identified by a specific proteomic technique per se. Instead, selected spots are submitted to mass spectrometry for identification. The identification is based on matching peptides from the protein samples excised from gels with peptides obtained from the theoretical digestion of all mouse proteins that are available from different databases. Thus, mass spectrometry did not allow us to distinguish between the constitutive (HSP70) and inducible (HSP72) forms of the protein as a specific antibody allows in IHC or western blotting. In the proteomic analysis, we have observed differences in the expression of the “HSP70 family”, without specifying which member or members of this family are differentially expressed (most likely HSP72), while when one specifically focuses on constitutive HSP70 in the western blot, no differences are observed for this protein.

Thus, we proceeded to perform a 2D western blot analysis (isoelectric point in one dimension vs. molecular weight in the second) for these proteins. In these conditions, we were able to find that the band observed in 1D WB for HSP70 is actually composed of two fractions, one of which was specifically expressed by the peri-infarct tissue (Fig. 4 and supplementary data 1) and absent in contra-lateral tissue.

Immunohistochemical studies with brain sections using a specific antibody for HSP72 corroborated this finding. As we can see in Fig. 5, HSP72 only is expressed in the peri-infarct tissue, while HSP70 is constitutively expressed in all brain tissues (lesion, peri-infarct and contra-lateral sections).

Our attempts to obtain similar results with the other differential proteins found in 2D-PAGE studies (DHRP-2, eIF2α, HSP27, Prohibitin or β-Tubulin) were unsuccessful. Although we were unable to find other qualitative differences in protein expression in the peri-infarct tissue apart from HSP70, we cannot rule out the possibility that some fractions of the other proteins could also specifically be expressed in this tissue, under other experimental conditions (use of different antibodies, and western blot or immunohistochemical experiments).

Our findings are in agreement with previously reported studies. Other authors have also shown that HSP70 is over-expressed in the aged or stressed brain^25^, in general, and particularly in the ischemic brain^10^,^11^,^12^, though none of these studies have specifically focused on the peri-infarct area. Additionally, there is a study that reports a global analysis of protein expression in a model of the ischemic penumbra, which described the up-regulation of several heat shock proteins^26^. However, this study was performed in an *in vitro* model, and its results may not be directly extrapolated to the *in vivo* situation. Other studies also describe the up-regulation of the HSP70 protein at the peri-infarct area, but none of them specifies that only the inducible form of this protein is specific for this region, which may mislead the performance of experimental studies when improper antibodies are selected (as in our 1D western blot analysis). Thus, in general, we believe that our global analysis of protein expression in the peri-infarct region adds important information to the current literature.

We are aware that our study presents a series of limitations. First, COFRADIC provided us with a list of 72 significant differences in protein expression from the peri-infarct tissue. However, we have focused our study on the proteins also observed in 2D-PAGE studies. Our intention has always been to perform a classical 2D-PAGE proteomic study and then to test the suitability of COFRADIC to complement and confirm gel-based studies. The data obtained with one animal brain are not statistically relevant. However, we believe that we have achieved our objective and demonstrated that COFRADIC is a good complement for 2D-PAGE, with some strong points in its favour, which encourages us to use COFRADIC more intensively in future studies. Having said that, we should always keep in mind that COFRADIC was tested in only one animal, and, therefore, the results derived from this approach are not conclusive.

In addition, we have found experimental conditions (including the selection of selective antibodies) to demonstrate that HSP72 is the only member of the heat shock 70 gene family that is specifically expressed at the peri-infarct tissue, while other proteins presented only quantitative but not qualitative differences in expression in the peri-infarct tissue. However, we cannot rule out the possibility that other proteins may also be specifically expressed in that region, which may require different experimental parameters than the ones tested here.

Finally, our study was focused on a particular time point after the induction of ischemia (48 h), with the aim to prevent an excessive influence of potential markers of inflammation and oedema (which are typically present in the brain during the acute phase of ischemia). Thus, the suitability of HSP72 as a biomarker for the peri-infarct tissue at different stages of the disease has yet to be proven.

In conclusion, after a global proteomic analysis of the ischemic brain by two different and complementary proteomic approaches, western blotting and immunohistochemistry, our data supports the conclusion that the inducible form of the 70 kDa Heat Shock Protein family (HSP72 or HSP70i) is an specific biomarker of the peri-infarct area, whereas the constitutive form of this family (HSC70 or HSP70c) is not specific for this area. Notice that we are interested in the presence of the protein in the peri-infarct tissue at the studied time-point, to be used for molecular recognition of that region. Thus, the exact role that the protein may be playing at that point, the type of cells that express the protein, differences between animal models and other related issues are out of the scope of our work.

## Methods

All procedures involving the use of research animals were approved by the Research Committee of the University Clinical Hospital of Santiago de Compostela (Spain) and were performed according to the “Principles of laboratory animal care” (NIH publication No. 86-23, revised 1985), as well as specific Spanish (RD 1201/2005 and RD 53/2013) and European Union (Directives 86/609/CEE, 2003/65/CE, 2010/63/EU) legislation.

Proteomic and western blotting studies were performed by a scientist blinded to brain tissue collection and processing.

### Animal model of permanent focal brain ischemia

A total of 14 adult male Sprague-Dawley rats (Harlan, Barcelona, Spain) weighing 300 ± 25 g were used in this study. Animals were anaesthetized with 3% Sevoflurane (Abbot Laboratories, Madrid, Spain) in a gas mixture of 70% N_2_ and 30% O_2_. Permanent focal cerebral ischemia was induced by ligature of the left common carotid artery (CCA) and of the ipsilateral distal middle cerebral artery (MCA), as previously described^27^. Animals were sacrificed 48 hours after surgery by an overdose (8%) of Sevoflurane, and the brains were extracted and processed for proteomic studies as indicated below. For immunohistochemistry, two of the animals were transcardially perfused with 100 ml of phosphate buffer solution (PBS) (Sigma-Aldrich, St Louis, MO) followed by 300 ml of 4% formaldehyde (Sigma-Aldrich, St Louis, MO) prior to brain extraction.

### Tissue preparation for proteomic analyses

Brains from nine ischemic rats were extracted, sliced in 2-mm-thick slices and stained with triphenyl tetrazolium chloride (TTC) (Sigma-Aldrich, St Louis, MO). TTC is a marker for metabolic activity and reliably indicates necrotic tissue (the infarct core) for up to 3 days after ischemia^28^. Following TTC exposure, the metabolically active tissue stains in red, while the necrotic tissue remains pale. The infarct core was excised. Then, the adjacent 2-mm-thick portion of the brain tissue surrounding the lesion core was also excised and was defined as the peri-infarct tissue^29^. Finally, a symmetrical portion of tissue corresponding to this stripe was extracted from the contra-lateral brain hemisphere, and used as the control tissue.

The peri-infarct and contra-lateral tissues were subjected to protein fractionation to separate proteins in three different fractions, membrane, soluble and insoluble proteins, using the ProteoExtract® Native Membrane Protein Extraction Kit (Calbiochem Merck KGaA, Darmstadt, Germany), according to the manufacturer’s instructions. The protein content of the three fractions of proteins was separately quantified by the Bradford method (Bio-Rad, Hercules, CA).

### Two-dimensional electrophoresis (2D-PAGE)

Contra-lateral and peri-infarct tissue samples from six rats were used for 2D gel electrophoresis. Thirty-six samples in total were prepared: (6 animals) × (2 samples per animal, peri-infarct and contra-lateral tissue) × (3 fractions of proteins per tissue, membrane, insoluble and soluble proteins). The proteins were separated by isoelectric point and, subsequently, by molecular weight, as previously described^30^. Protein patterns from 72 gels (two duplicates per sample) were analysed using the PDQuest 7.0 software from Bio-Rad. This software discriminates statistically significant differences (p < 0.05) for protein expression levels between samples.

Brains from three additional animals were processed to obtain gels stained with 0.1% Coomassie Brilliant Blue R-250 (Bio-Rad, Hercules, CA) for mass spectrometry. Protein spots of interest were manually excised and digested according to the procedure described by Shevchenko *et al.*^31^ for protein identification by mass spectrometry. MALDI-TOF mass spectrometry analyses were performed using a 4700 Proteomics Analyzer MALDI TOF/TOF mass spectrometer (Applied Biosystems, Carlsbad, California). Peptide mass fingerprints were used for protein identification. The non-redundant NCBI and SwissProt databases were searched using MASCOT 1.9 (matrixscience.com) through the Global Protein Server v3.5 from Applied Biosystems. The search parameters were: carbamidomethylated cysteine as the fixed modification, oxidized methionine as the variable modification, peptide mass tolerance 50 ppm, and one missed trypsin cleavage site.

### COFRADIC (combined fractional diagonal chromatography) analysis

Protein expression differences between the contra-lateral and peri-infarct regions from one rat were analysed by the methionine COFRADIC gel-free proteomics technique^22^,^32^. For this purpose, proteins were digested overnight at 37 °C with 5 μg of endoproteinase lys-C (Roche Diagnostics, Vilvoorde Belgium), peptides were *N*-propionylated with either ^12^C_3_- or ^13^C_3-_propionate^23^, and methionine-containing peptides were isolated as described^23^. Fractions containing these methionyl peptides were analysed by LC-MS/MS using an Ultimate 3000 HPLC system (Dionex, Amsterdam, The Netherlands) connected in-line to a LTQ Orbitrap XL mass spectrometer (ThermoScientific, Bremen, Germany).

The mass spectrometer was operated in top 6 data-dependent mode and full scan MS spectra were acquired with a resolution of 60,000 at m/z 400. From the MS/MS data in each LC-MS/MS run, Mascot generic files (mgf) were created using the Mascot Distiller software (version 2.2.1.0, Matrix Science). The resulting mgf files were searched against the SwissProt database and restricted to rat proteins. Endo-LysC was set the protease in Mascot, allowing for one missed cleavage. Only oxidation of methionine was set as a fixed modification, whereas pyro-glutamate formation and acetylation of N-terminus were set as variable modifications. Tolerances for the precursor ion mass and fragment ions masses were set to ±10 ppm and 0.5 Da, respectively. Determination of the light (^12^C_3_) and heavy (^13^C_3_) propionate labelled peptides was established for further quantification using the quantization option in Mascot. Mascot Distiller was used to quantify protein expression differences between peri-infarct and contra-lateral regions. Only proteins that were identified and quantified by at least two different peptides were withheld. Protein expression was considered quantitatively different when the peri-infarct/contra-lateral ratio was >3 or <0.33, (this value includes statistically differences with p <0.05).

### Western blot analysis

Samples from two animals were used to validate the proteomics results by one- and two-dimensional western blotting. For this purpose, samples were homogenized and fractionated into membrane, soluble and insoluble proteins, as described above.

Samples for 1D western blot were diluted 1:1 with Laemmli buffer (Bio-Rad, Hercules, CA) and 40 μg of protein were loaded on 10% SDS-PAGE gels. Samples for 2D western blots (40 μg of protein) were loaded in 200 μl of rehydration buffer, and proteins were separated by isoelectric point and then by molecular weight, as previously described^30^.

Proteins from one or two-dimensional gels were transferred onto a low fluorescence PVDF membrane (Millipore, Billerica, MA) in semi-dry conditions at 15 V for 45 minutes, blocked in 5% fat-free powder milk for 2 hours at room temperature, and incubated overnight with mouse monoclonal anti-rat HSP70 antibody (1:1000, Abcam, Cambridge, MA). Membranes were simultaneously incubated with a rabbit polyclonal anti-rat β-Actin (1:3000, Abcam, Cambridge, MA). For detection, either a goat anti-mouse Cy3-labelled secondary antibody (1:3000, GE, Barcelona, Spain) or a goat anti-rabbit Cy5 (1:3000, GE, Barcelona, Spain) were used, and images were acquired using a Molecular Imager FX Pro-plus (BioRad, Hercules, CA) and analysed with QuantityOne or PDQuest software.

### Immunohistochemistry (IHC)

Brains from two animals were used for IHC analysis of HSP70. Brains were sliced in 3 mm-thick sections, post-fixed in 4% formaldehyde overnight and further immersed in 20% sucrose for 24 hours. Brain slices were embedded in OCT (Sakura Finetek Inc., Torrance, CA) and stored at –80 °C until use. Ten-micron (10 μm) coronal sections were obtained using a cryostat (Tissue-Tek Cryo_3_, Sakura Finetek Inc.). The sections were incubated with 3% H_2_O_2_ and 10% methanol in PBS to block endogenous peroxidases, and with 3% normal serum and 0.2% Triton X-100 in PBS to block non-specific binding sites. The sections were subsequently incubated with a primary antibody against the inducible form of the Hsp70 protein (SPA-810, 1:50 dilution Stressgen Biotechnologies Corp., Victoria BC, Canada) or against both the constitutive and inducible forms of the Hsp70 protein (ab8439, 1:50 dilution Abcam, Cambridge, MA) for 1 hour, and with a biotin-conjugated secondary anti-rabbit antibody (1:200; Vector Laboratories Inc., Burlingame) and streptavidin-conjugated peroxidase (Vecstatin Abc kit, Vector Laboratories Inc.). Colour was developed by the addition of DAB (Dako, Glostrup, Denmark). To evaluate the background reaction, procedures were also performed on sections incubated with only the secondary antibodies (indirect technique). The stained slices were visualized under an IX-51 microscope (Olympus Life and Material Science Europe GMBH, Hamburg, Germany) attached to a DS-U2 LCD camera (Nikon Instruments Inc., Melville, NY).

### Statistical Analysis

The data are presented in the text as the mean ± standard deviation, unless otherwise stated. Correlation coefficients for reproducibility among gels in 2D-PAGE studies were calculated with the PDQuest 7.0 software (Bio-Rad, Hercules, CA). The mean intensity of each spot from the control sample (contra-lateral tissue) was plotted against the mean intensity of each spot from the target sample (peri-infarct tissue). The experimental data are fitted to a straight line, and the correlation coefficient (r^2^) is provided by the software. This procedure was individually performed for the three fractions of proteins (soluble, insoluble and membrane).

## Additional Information

**How to cite this article**: Brea, D. *et al.* Study of Protein Expresion in Peri-Infarct Tissue after Cerebral Ischemia. *Sci. Rep.* **5**, 12030; doi: 10.1038/srep12030 (2015).

## Supplementary Material

Supplementary Information
