# Supplementary material for: Study of Protein Expresion in Peri-Infarct Tissue after Cerebral Ischemia
Source: Sci Rep. 2015 Jul 8;5:12030. doi: 10.1038/srep12030 (PMC4495553; doi:10.1038/srep12030)

**STUDY OF PROTEIN EXPRESION IN PERI-INFARCT TISSUE AFTER CEREBRAL ISCHEMIA**

*David Brea,^1, 2^ Jesús Agulla,^1, 3^ An Staes,^4, 5^ Kris Gevaert,^4, 5^ Francisco Campos,^1^ Tomás Sobrino,^1^ Miguel Blanco,^1^ Antoni Dávalos,^2^ José Castillo,^1^ and Pedro Ramos-Cabrer^1, *^*

^1^Neurology Department, Neurovascular Area, Clinical Neurosciences Research Laboratory, University Clinical Hospital, Health Research Institute of Santiago de Compostela (IDIS), University of Santiago de Compostela, Spain.

^2^Cellular and Molecular Neurobiology Research Group and Grup de Recerça en Neurociencies del IGTP, Department of Neurosciences, Fundació Institut d'Investigació en Ciències de la Salut Germans Trias I Pujol-Universitat Autónoma de Barcelona, Badalona, Spain.

^3^Research Unit, University Hospital of Salamanca and Institute of Health Sciences of Castilla and Leon, Salamanca, Spain.

^4^ Department of Medical Protein Research, VIB, Ghent, Belgium

^5^ Department of Biochemistry, Ghent University, Ghent, Belgium

***Correspondence to**: Dr. Pedro Ramos-Cabrer (pramos@cicbiomagune.es)

Molecular Imaging Unit. CIC biomaGUNE. 20009 Paseo de Miramón 182, Donostia - San Sebastián, Spain. Telephone: (34) 943005426. Fax: (34) 943005001

**Supplementary data 1**

Western blots shown in figure 4 are cropped parts of the total gels, for better magnification and visual presentation. Complete western-blots are presented here.

All gels were performed in the same experimental conditions, as described in the materials and methods section of our work.

**1D Western blots**


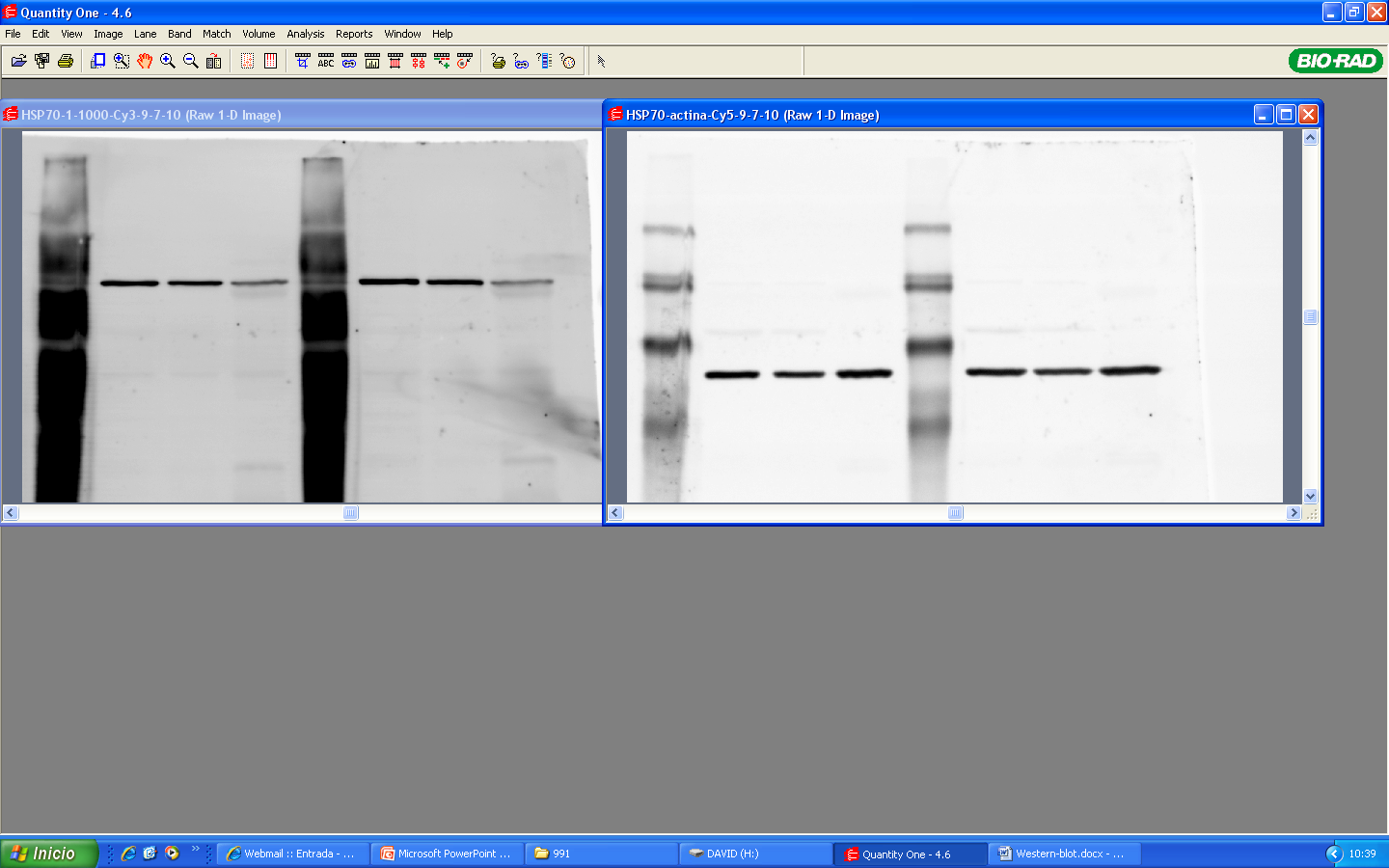


Left ,HSP70 bands (2 duplicates ). Right, β-actin bands (2 duplicates).

Cropped and magnified regions, as presented in figure 4, are shown next:


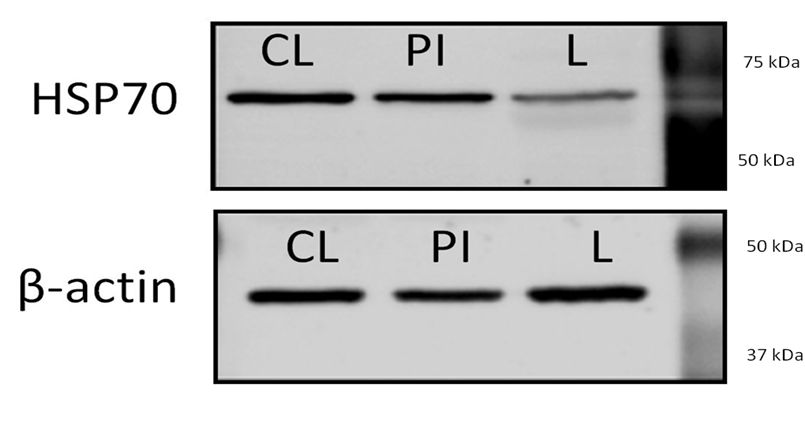


**2D Western blots**

Membranes for 2D gels are usually big, and it is a usual practice to crop them to reduce their size to a specific target regions (for predefined isoelectric point and molecular weight ranges) to save the use of large amounts of expensive antibodies. Therefore, and according to literature [J. Exp. Biol 205, 273–278 (2002) and J. Biol. Chem. 273(45), 29857–29863 (1998)], gels where cut before scanning at the regions where we expected HSP70 proteins to be located. That is, in the region of isoelectric Point (PI) between 5-6 and in the region of 70 kDa of molecular weight. Therefore membranes were cut according to these values. The same strategy was followed for the detection of β-actin, cutting the membranes between a PI. of 4-6, and molecular weight region between 50 and 37 KDa.


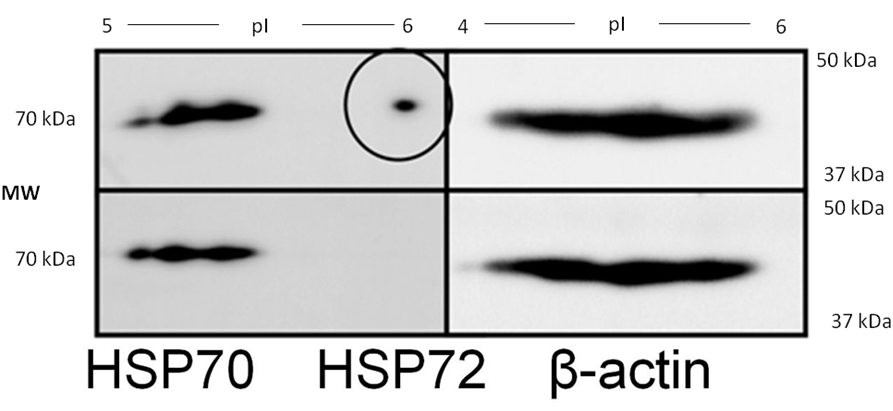

Supplement: Supplementary Information [file srep12030-s1.doc]
